# Traumatic Isolated Right Lobe Devascularization of the Liver: An Unusual Case

**DOI:** 10.7759/cureus.40621

**Published:** 2023-06-19

**Authors:** Mehak Sehgal, Teg R Singh, Devendra Yadav, Anjan Dhua, Minu Bajpai

**Affiliations:** 1 Pediatric Surgery, All India Institute of Medical Sciences, New Delhi, IND

**Keywords:** major trauma, nom, non-operative management, paediatric liver trauma, isolated devascularization

## Abstract

Isolated liver lobe devascularization is a very rare case, with conflicting literature regarding management. We describe a very unusual case of traumatic isolated right lobe devascularization of the liver with its attendant management challenges. An eight-year-old boy with a history of road traffic accidents presented with abdominal pain. Although the child was hemodynamically stable on presentation, extended focused assessment with sonography in trauma was positive. Contrast-enhanced computed tomography (CECT) scan of the torso revealed a nonenhancing right lobe of the liver involving segments 5-8 and the gross hemoperitoneum. Nonoperative management was tried. There were persistent high-grade fever spikes, for which prophylactic antibiotics were started, but the fever workup was negative. Abdominal drains were inserted to drain fluid and relieve distress. Output was noted to be bilious on day 21 of injury. Diagnostic laparoscopy on day 22 revealed hypertrophied left lobe of the liver with an absent (autolyzed) right lobe. The subsequent ward course was uneventful, and the child was discharged in stable condition. Thus, the indication of surgery in such cases is clinical deterioration, not radiological findings. Management should be in a dedicated trauma center with immediate operating room availability.

## Introduction

The liver and spleen are the commonly injured organs in blunt abdominal trauma [1-3]. The increased use of contrast-enhanced computed tomography (CECT) of the torso has resulted in an enhanced and accurate diagnosis of minor-grade injuries, which has led to a better understanding of the course of trauma on the child's physiology. While most children recover uneventfully, the mortality rate can be significant in higher-grade (IV and V) injuries if not properly managed [4]. The resultant advent of nonoperative management (NOM) [5,6] resulted in excellent rates of survival without the added morbidity of laparotomy and anesthesia.

Isolated devascularization of a hepatic lobe, without any associated hollow viscus or solid organ injury, is a rare presentation of abdominal trauma. Only a few anecdotal case series and reports address the topic, and all advocate early surgical intervention [7-9]. We present a case of right hepatic lobe devascularization following blunt trauma to the abdomen in an eight-year-old child and the challenges faced in the nonoperative management of the same.

## Case presentation

An eight-year-old boy, with no known premorbid medical history, presented to our trauma center with an alleged history of fall and subsequent skid from a running two-wheeler, 12 hours post-injury. The child had a history of severe abdominal pain with abdominal distension post-trauma. Although there was a history of loss of consciousness, there was no vomiting, seizure, chest pain, or hematuria. The initial assessment and resuscitation were done elsewhere.

On arrival in the emergency department, the airway was patent and the child was maintaining saturation on room air. The blood pressure and pulse rate were within normal centiles for age. Although the sonological pneumo-scan was negative, Focused Assessment with Sonography for Trauma (FAST) showed gross hemoperitoneum. On a per-abdomen exam, guarding and tenderness were noted in the right hypochondrium. X-ray examinations of the chest and pelvis showed no abnormality.

**Figure 1:**
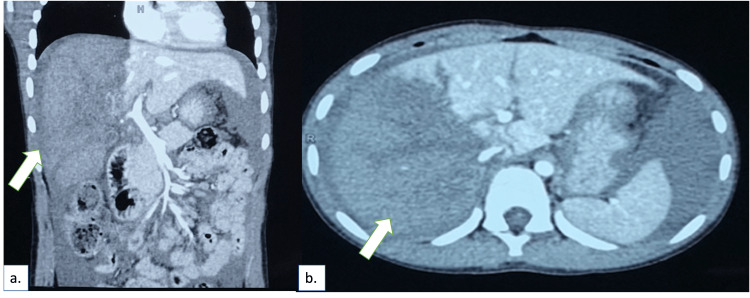
Contrast-enhanced computed tomography done at admission showing nonenhancing right lobe (arrow) with hemoperitoneum: (a) coronal section; (b) axial section.

CECT scan of the chest and abdomen showed a normal study of the thorax, but the right lobe of the liver showed nonenhancement in all four segments. A laceration was also noted in segments 4a and 4b of the liver. The right portal vein was seen, but distal posterior and anterior branches were not visualized. The right branch of the hepatic artery was also not visualized. A focal collection of contrast in the periportal region was observed, with a gross hemoperitoneum. No signs of other solid organ injury or hollow viscus injury were noted (Figure 1). 

Given the hemodynamically stable condition, a decision was taken to manage the patient conservatively, with close monitoring, keeping a low threshold for surgical intervention.

**Figure 2:**
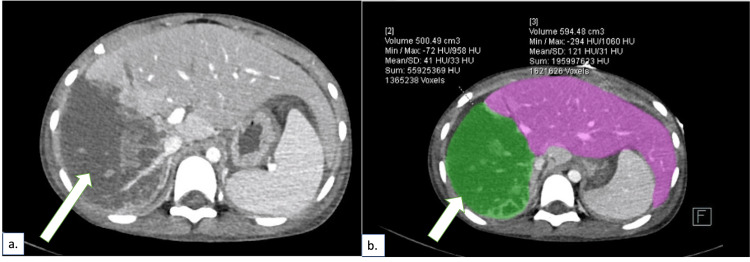
Contrast-enhanced computed tomography on post-injury day 5 showing a nonenhancing right lobe (arrow) similar to that at presentation (Figure 1): (a) axial section; (b) volumetric analysis of the involved area of the liver (green).

Ultrasound scans were done on post-admission day 3, which showed a heterogenous right lobe of the liver with no obvious flow in the right portal vein, and moderate free fluid. No pseudoaneurysm or abscess was seen. The patient, however, developed significant respiratory distress on post-admission day 5. A chest X-ray was done, which revealed bilateral moderate to massive pleural effusions, necessitating drainage. A CECT scan was again performed, which showed a similar picture (Figure 2).

High-grade fever spikes complicated the admission course, but fever workup, including cultures from blood, urine, pleural fluid, and ascitic tap, revealed no growth. High-grade antibiotics were started prophylactically. Given gradually increasing abdominal girth, multiple fever spikes, and persistent tachycardia and tachypnoea, a pigtail catheter was placed in the right iliac fossa on post-admission day 15. In the absence of an identifiable cause, autolysis of the devascularized liver with associated inflammatory response was retrospectively deemed as the cause for the clinical symptoms.

Pigtailed catheter output decreased the second day after insertion: however, because of suspected bilious output from the drain on admission day 21, the child was taken up for diagnostic laparoscopy. Intraoperatively, loculated fluid collection in the abdomen and interbowel adhesions were observed. The left lobe of the liver was hypertrophied, and the right lobe was not visualized; it was replaced by omentum and small bowel loops, which were not disturbed. Minimal adhesiolysis was done, and two wide bore drains were placed.

The postoperative course was uneventful, with output from both drains gradually reducing to nil. No bile leak or purulent discharge was ever observed from the abdominal drain. Both drains were removed on postoperative day 8. The patient was discharged in a stable condition, without fever, and was accepting full orals. Liver function tests of the patient remained unimpaired throughout the admission course.

## Discussion

It is well-known that patients with higher grades of liver injury (IV and V) are prone to complications, and the risk of morbidity as well as mortality is higher [4]. Nonoperative management is the preferred approach in most cases, with brilliant survival rates (90%) and an acceptable rate of complications (<4%). Successful nonoperative therapy is proven to have lower transfusion requirements, abdominal infections, and hospital lengths of stay [5]. This is particularly relevant in cases of liver trauma. Unlike the spleen, liver-related bleeding is morbid and can be made worse by operative intervention, and manipulation of venous injuries can result in massive hemorrhage and death [10,11].

However, this approach may be called into question in cases with major disruption to vascular inflow to the liver, resulting in large devascularized areas, prone to necrosis, bleeding, infection, and bile leak, among a host of other complications. Some authors found no increase in mortality in patients that failed initial nonoperative management [12], while analysis by others has revealed a higher mortality rate in patients who undergo subsequent surgery [13]. The importance of the initial management decision in patients with high-grade liver injuries is, thus, paramount.

Major vascular disruption to a lobe of the liver, resulting in major liver necrosis, can be seen after angioembolization for an active bleed or rarely following abdominal trauma. The majority of the studies would advocate early surgical intervention, before the onset of complications [14,15]; however, the risk of liver resection in such a precarious situation cannot be ignored.

Consequences of necrosis, including but not limited to elevated liver transaminases, coagulopathy, bile leak, abdominal pain, feeding intolerance, respiratory compromise, renal failure, and sepsis, should always be kept in mind, and close monitoring should be done in these patients. Diagnosis of major liver necrosis is on a follow-up CT scan: persistent devascularization of a suspected intraparenchymal hemorrhage and/or air pockets may indicate the same.

The devascularized right lobe of the liver in our patient autolyzed on its own and required no operative intervention. Diagnostic laparoscopy on admission day 22 revealed complete resorption of the same. The recurrent fever spikes in our case could be attributed to the major remodeling process going on inside the body. Repeated cultures from various sources showed no microbial growth and ultrasound examination was negative for any purulent collection. The patient did require supportive management in the form of drainage of chest fluid and ascitic fluid, to relieve distress as and when necessary.

## Conclusions

Isolated liver lobe devascularization is rare, with conflicting literature on management. There is role of conservative management and diagnostic laparoscopy in these patients, which avoids the morbidity of a laparotomy. Furthermore, drain placement facilitates early recovery of the patient. The indication of surgery is clinical deterioration and not CT findings. Management of such cases should be in a dedicated trauma center with immediate operating room availability.
